# Positional information in axolotl and mouse limb extracellular matrix is mediated via heparan sulfate and fibroblast growth factor during limb regeneration in the axolotl (*Ambystoma mexicanum*)

**DOI:** 10.1002/reg2.40

**Published:** 2015-10-12

**Authors:** Anne Q. Phan, Jangwoo Lee, Michelle Oei, Craig Flath, Caitlyn Hwe, Rachele Mariano, Tiffany Vu, Cynthia Shu, Andrew Dinh, Jennifer Simkin, Ken Muneoka, Susan V. Bryant, David M. Gardiner

**Affiliations:** ^1^ Department of Developmental and Cell BiologyUniversity of California IrvineIrvineCalifornia92697‐2305USA; ^2^ Department of Cell and Molecular BiologyTulane UniversityNew OrleansLouisiana70118,USA

**Keywords:** axolotl, ECM, heparan sulfate, pattern formation, positional information

## Abstract

Urodele amphibians are unique among adult vertebrates in their ability to regenerate complex body structures after traumatic injury. In salamander regeneration, the cells maintain a memory of their original position and use this positional information to recreate the missing pattern. We used an in vivo gain‐of‐function assay to determine whether components of the extracellular matrix (ECM) have positional information required to induce formation of new limb pattern during regeneration. We discovered that salamander limb ECM has a position‐specific ability to either inhibit regeneration or induce de novo limb structure, and that this difference is dependent on heparan sulfates that are associated with differential expression of heparan sulfate sulfotransferases. We also discovered that an artificial ECM containing only heparan sulfate was sufficient to induce de novo limb pattern in salamander limb regeneration. Finally, ECM from mouse limbs is capable of inducing limb pattern in axolotl blastemas in a position‐specific, developmental‐stage‐specific, and heparan sulfate‐dependent manner. This study demonstrates a mechanism for positional information in regeneration and establishes a crucial functional link between salamander regeneration and mammals.

## Introduction

Limb regeneration is the outcome of a complex sequence of events mediated by interactions between the cells that arise from the tissues of the amputated stump. There is much to be discovered before regeneration can be induced in humans. In addition to being able to recruit all the appropriate cell types to reform the missing structure, it will be necessary to learn how to orchestrate the behavior of these cells in order to have the cells remake the appropriate pattern of the limb (Endo et al. 2004). Much is being learned from stem cell biology about how to expand and differentiate multipotent cells that can give rise to various tissues. The next challenge then will be to ensure that these cells form tissues that have the appropriate spatial patterns relative to each other so as to restore organ function (McCusker & Gardiner 2014). For example, the muscles and tendons have to span joints in order for the limb to move. Thus achieving successful regeneration in humans will require an understanding of how to provide the necessary cues in the adult environment to regenerate pattern and function.

Our understanding of the mechanisms of pattern formation during regeneration has not advanced in recent years as dramatically as our understanding of the mechanisms regulating differentiation (developmental genetics and stem cell biology) and dedifferentiation (induced pluripotent stem cell biology). The phenomenology of positional information is well understood based on earlier studies, including the determination that the cells that encode positional information are localized in the loose connective tissues of the limb (French et al. 1976; Bryant et al. 1981, 2002; Rinn et al. 2006; Nacu et al. 2013). The way in which cells use positional information to regenerate the limb pattern was formally conceptualized by the polar coordinate model (French et al. 1976; Bryant et al. 1981) such that cells have a positional code with respect to the three limb axes: proximal/distal, anterior/posterior, and dorsal/ventral. When cells with different positional codes (i.e., from different positions relative to the limb axes) interact with each other, they are induced to proliferate and their progeny acquire new positional information that is required to replace the missing parts of the pattern. This process of “intercalation” is reiterated until all the missing pattern is restored and regeneration is completed (French et al. 1976; Bryant et al. 1981; Nakamura et al. 2007).

Positional information is localized to the loose connective tissue of the limb, which is composed of both cells and a biochemically complex extracellular matrix (ECM). Since the connective tissue cells are responsible for synthesis of the ECM, we hypothesized that positional information is stably encoded in the ECM by these cells. Although this information is stable, the cells acquire new positional information during regeneration (French et al. 1976; Bryant et al. 1981; McCusker & Gardiner 2013; Roensch et al. 2013), and subsequently encode this new information when they resynthesize the new ECM. The ability of heparan sulfate in the ECM to mediate cell−cell signaling has been demonstrated for a number of signaling pathways important in regeneration and pattern formation including fibroblast growth factor (FGF), shh, BMP, TGF‐β, and Wnt (Rapraeger et al. 1991; Yayon et al. 1991; Olwin & Rapraeger 1992; Häcker et al. 2005). We therefore also hypothesized that the ability of cells to encode position‐specific information in the ECM is dependent on position‐specific modification of heparan sulfate.

**Figure 1:**
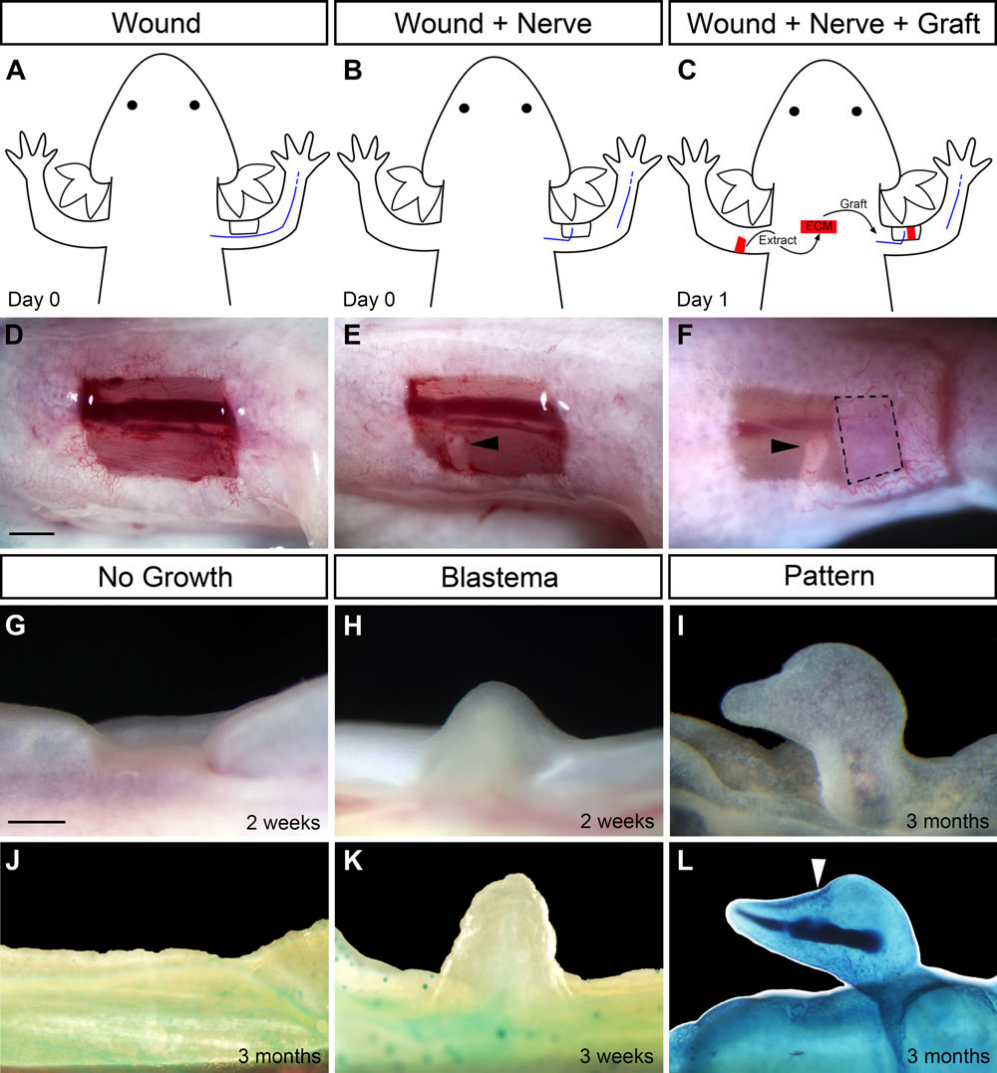
The accessory limb model as a gain‐of‐function assay for blastema formation and pattern formation. (A), (D) A full‐thickness skin wound was made on the anterior region of the upper arm. (B), (E) The brachial nerve was severed at the elbow and deviated to the wound site which resulted in the formation of an ectopic blastema (H). (C), (F) Grafts of extracellular matrix (ECM) were placed in the wound site to assay for pattern formation. Observed outcomes of the assay are wound healing with “No Growth” of a blastema (G, J), “Blastema” but no pattern (H, K) or blastema with “Pattern” (I, L). (J), (K), (L) Appearance of samples after preparation to visualize skeletal patterns in whole‐mount preparations. Dashed lines (F) outline the ECM graft. Black arrowheads (E, F) indicate the severed end of the deviated nerve. White arrowhead (L) indicates the position of the joint of the induced digit‐like structure. Scale bar 1 mm.

To determine whether or not the ECM encodes positional information, we took advantage of the accessory limb model (ALM), which is a gain‐of‐function assay for signaling that regulates blastema formation and positional information (Endo et al. 2004; Satoh et al. 2007). In this assay, an ectopic blastema is induced on the side of the arm by making a small full‐thickness skin wound and surgically deviating the brachial nerve to the wound site (Fig. 1A, B, D, E). Within a few days post‐surgery an ectopic blastema develops and grows; however, without further signaling, it does not form ectopic limb structures and eventually reintegrates into the limb (Endo et al. 2004; Satoh et al. 2007). The ectopic blastema can be induced to form an ectopic limb if positional information is provided from the side of the limb that is opposite to the position of the surgically created wound. This is done by grafting a piece of skin (epidermis plus dermis) from the opposite side of the limb (e.g., posterior skin grafted to an anterior wound with a deviated nerve) (Endo et al. 2004). Here we report the results of experiments designed to discover the minimal components of skin grafts that are capable of stimulating the formation of ectopic limb structures. Since the grafted skin with posterior positional information contains both cells and ECM, we decellularized anterior and posterior donor skin prior to grafting into wounds with deviated nerves in order to determine whether the ECM encodes positional information (Fig. 1C, F).

We discovered that anterior and posterior ECMs have position‐specific signaling properties that either inhibit regeneration or induce de novo limb structure, and that this difference is mediated by growth‐factor‐interacting heparan sulfate in the ECM. In addition, we found evidence that the position‐specific functionality of the ECM arises as a consequence of position‐specific differential expression of heparan sulfate sulfotransferases during regeneration. Most importantly, the ability to assay ECM rather than cells allowed us to expand our study to include mammals in a way that has not been possible previously. By grafting mouse limb skin ECM into the axolotl ALM, we discovered that mammalian ECM can inhibit blastema formation, or induce limb skeletal pattern, or have no effect on regeneration depending on the position around the limb circumference from which the graft is derived. As with axolotl ECM grafts, the ability of the mouse ECM to provide position‐specific signaling was dependent on the presence of heparan sulfate. Finally, we found that the spatial−temporal variation of positional signaling in mouse limb skin ECM is regulated by differential expression of specific heparan sulfate sulfotransferases that modify the sulfation patterns of heparan sulfates in the ECM. This study demonstrates a novel mechanism for positional information in regeneration and establishes a crucial functional link between regeneration in salamanders and mammals.

### Results

#### Cell‐free ECM derived from different positions around the limb circumference can both inhibit and enhance ectopic limb regeneration

Previous studies have demonstrated that formation of an ectopic limb on the anterior side of an axolotl limb requires a graft of connective tissue derived from the posterior side of the limb (Endo et al. 2004). These studies have involved the grafting of full‐thickness skin that included cells as well as the ECM, and thus both posterior (graft‐derived) and anterior (host‐derived) cells participated in regeneration of the de novo limb. Without a posterior skin graft, an ectopic blastema can be induced to form that is derived only from anterior cells, but it does not progress to form an ectopic limb (Endo et al. 2004; Satoh et al. 2007; this paper). These findings have led to the conclusion that although anterior cells can be induced to form regeneration‐competent blastema cells, they need to interact with cells with posterior positional information in order to regenerate an ectopic limb (Endo et al. 2004). Since a posterior skin graft that supplies the necessary posterior information contains both cells and ECM, we grafted decellularized posterior skin into an anterior wound to test whether or not posterior positional information is present in the ECM and whether it can induce anterior cells to form limb pattern.

Treatment of axolotl limb skin with urea kills the cells and subsequent washing removes water‐soluble cellular components. Macromolecules associated with the ECM or the lipid membranes remain associated with the ECM graft (e.g., GAGs and any bound factors) (Gilbert et al. 2006). As detailed below, we discovered that this urea‐treated ECM preparation had considerable biological activity, and that when relatively crude ECM was treated further to remove biologically active molecules (e.g., by enzyme treatment or washing with detergent and high concentrations of NaCl) this activity was lost.

Full‐thickness skin wounds on the side of an axolotl arm exhibited a range of regenerative responses depending on how the wounds were treated (Endo et al. 2004; Satoh et al. 2007; this paper). If the wound was allowed to heal without further manipulation (Fig. 1A, D), the wound healed without forming an ectopic blastema (“No Growth”; Fig. 1G, J). As described below, we sometimes observed a similar phenotype in response to grafted ECM (“No Growth”). If the brachial nerve was severed distally and surgically deviated to the site of the wound (Fig. 1B, E), an ectopic blastema that is equivalent to an amputation‐induced blastema formed (“Blastema”; Fig. 1H, K). These blastemas increased in size over a period of 2−3 weeks, but then began to regress without forming ectopic limb structures (Endo et al. 2004). If a graft of full‐thickness skin from the side of the arm opposite to the wound was grafted into the site of the wound/deviated nerve, a well‐patterned ectopic limb formed (Endo et al. 2004; Satoh et al. 2007). Although variable in the amount and complexity of the new limb pattern, these ectopic limbs can be remarkably normal in terms of their pattern (not illustrated) (Endo et al. 2004). In the present study, a similar procedure was used to graft decellularized ECM from the side of the arm opposite to the wound (Fig. 1C, F) to assay for the induction of ectopic limb skeletal elements (“Pattern”; Fig. 1I, L). We focused on anterior wounds because, due to the anatomy of the limb, nerves or grafts placed in posterior wounds often became dislodged when the animal began swimming.

**Figure 2:**
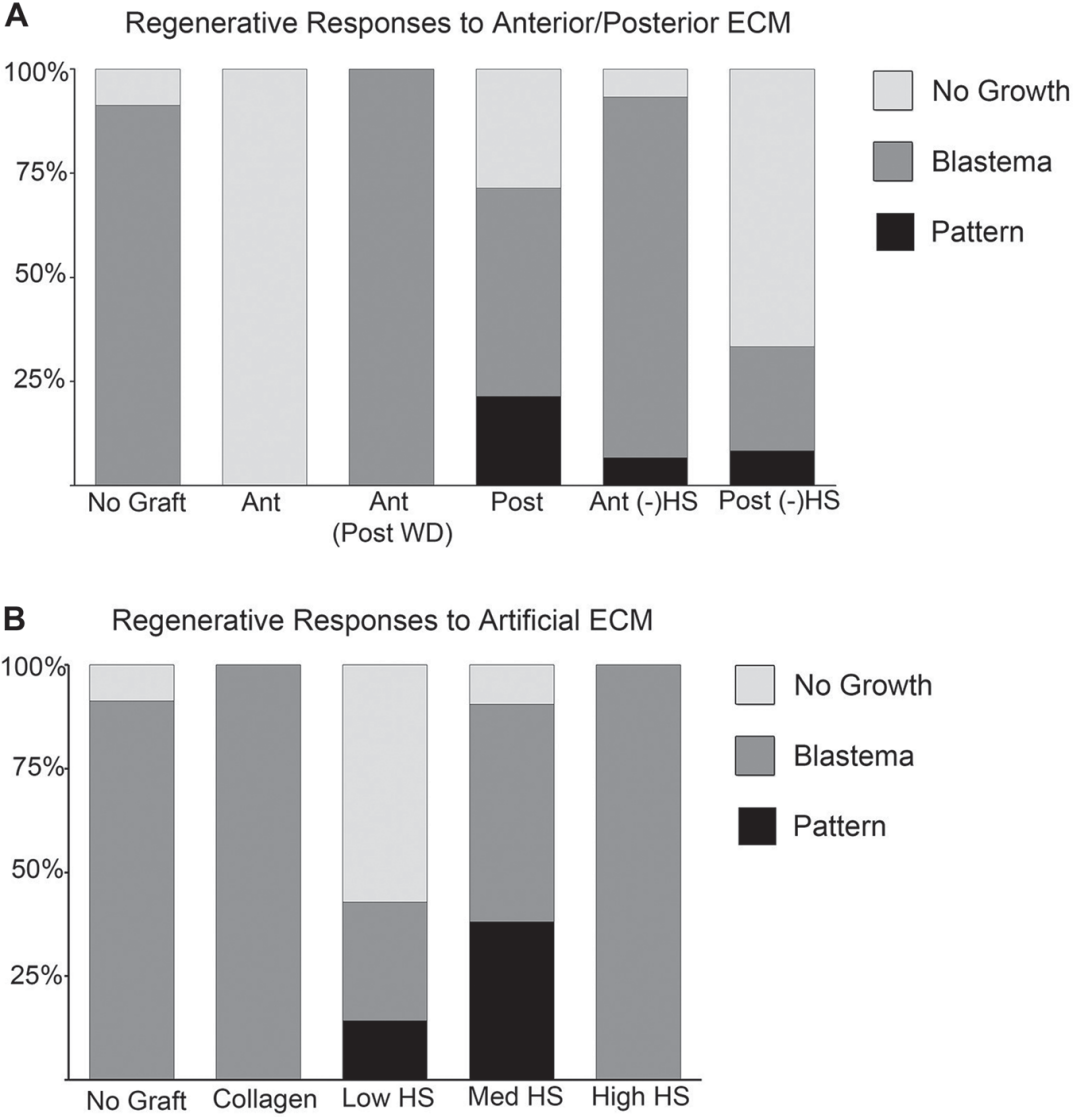
Heparan sulfate (HS) mediate position‐specific regulation of blastema formation and pattern formation during regeneration. (A) Differential regenerative responses to grafts of anterior/posterior extracellular matrix (ECM) with or without heparin lyase III (HepIII) treatment: Ant, anterior; Post, posterior; (−)HS, HepIII treated to remove HS from the ECM. (B) Dose‐dependent induction of pattern formation by grafts of artificial ECM containing low HS (2.5 mg/mL), med HS (5 mg/mL) or high HS (10 mg/mL) (Sigma, porcine intestinal mucosa).

Anterior wounds with a deviated nerve responded differently to grafts of anterior ECM or posterior ECM (Table 1; Fig. 2A). As reported previously (Endo et al. 2004), nerve‐deviated anterior wounds without an ECM graft formed ectopic blastemas at a high frequency, but did not form any pattern (Table 1; Figs 1H, K, 2A). In response to a graft of posterior ECM, some nerve‐induced ectopic blastemas (30%) formed ectopic cartilaginous structures that were morphologically similar to a distal phalangeal element (Table 1; Figs 1I, L, 2A). These structures were elongated, tapered at the tip and appeared to form a joint (Fig. 1L, arrowhead). In all cases, when we observed ECM‐induced pattern formation, it was less complete (hypomorphic) than what was typically observed in response to posterior skin grafts (ECM plus dermal cells) (Endo et al. 2004), even though these skin‐grafted ALM limbs are often hypomorphic (see Satoh et al. 2010). Surprisingly, grafts of anterior ECM inhibited blastema formation in all cases (Table 1; Fig. 2A). We also grafted anterior ECM to wounds on the posterior side of the arm. In all cases (*n* = 5, Fig. 2A, “Post WD”), ectopic blastemas without pattern formed, which is the same response as observed when ectopic blastemas are induced without an ECM graft (Table 1, Figs 1H, K, 2A). Aside from this one experiment involving grafting ECM to a posterior wound site, all grafting involved the response of cells in an anterior wound to signaling associated with grafted ECM. We thus observed a differential response of anterior limb cells to anterior ECM compared to posterior ECM (*P* < 0.001) as well as a differential ability of anterior ECM to inhibit regeneration from anterior wounds but not from posterior wounds (*P* < 0.001). We interpret this difference in both signaling and response to indicate that the ECM encodes position‐specific information that is involved in the regulation of regeneration.

**Table 1:** Position‐specific effects of anterior/posterior ECM grafts on blastema formation are dependent on heparan sulfate

Graft type	Total	Blastema*	Pattern**
No ECM graft	23	21 (91%)	0
Anterior ECM	8	0	0
Anterior ECM grafted in posterior wound	5	5 (100%)	0
Posterior ECM	14	10 (71%)	3 (30%)
Heparin lyase III treated anterior ECM	15	14 (93%)	1 (7%)
Heparin lyase III treated posterior ECM	12	4 (33%)	1 (25%)

*Percentage of total number of grafts that developed an ectopic blastema; **percentage of ectopic blastemas that developed skeletal elements with pattern.

#### Position‐specific information in the ECM is dependent on heparan sulfate

Growth factor signaling is important in the initiation and progression of axolotl limb regeneration (Mullen et al. 1996; Christen & Slack 1997; Christensen et al. 2001; Han et al. 2001; Satoh et al. 2008), and heparan sulfate in the ECM are known co‐factors required for growth factor signaling (Rapraeger et al. 1991; Yayon et al. 1991; Olwin & Rapraeger 1992). We therefore tested the hypothesis that heparan sulfate function to encode the position‐specific information in anterior versus posterior ECM. We selectively cleaved heparan sulfate from the ECM by treatment with heparin lyase III (HepIII, E.C. 4.2.2.8) prior to grafting anterior or posterior ECM into an anterior nerve‐deviated wound as described above. Anterior ECM grafts failed to inhibit regeneration after HepIII treatment (*P* < 0.001), and in most cases the nerve‐deviated wounds responded the same as if no ECM had been grafted (Table 1, Fig. 2A). Of the 14 blastemas that formed, one also formed pattern; however, given the low frequency of this response, it is unclear whether removal of heparan sulfate from anterior ECM was sufficient to induce pattern formation. The frequency of ectopic blastema formation decreased about 50% when posterior ECM was treated with HepIII (Table 1; Fig. 2A; 33 % compared to 71%; *P* = 0.11) and only one of the ectopic blastemas formed pattern. Given that only four ectopic blastemas formed, it is unclear whether the ability of posterior ECM to induce pattern formation in anterior ectopic blastemas was dependent on heparan sulfate. Since ectopic blastemas almost always formed in response to nerve deviation (e.g., 90%; Endo et al. 2004 and this study), the observed decrease in blastema formation when posterior ECM grafts were treated with HepIII (only 4 of 12) is noteworthy but not statistically significant (*P* = 0.11), and cannot be explained based on the available data. Nevertheless, the differential response to HepIII treatment of anterior ECM is consistent with the hypothesis that heparan sulfate in the ECM are involved in the encoding of position‐specific information.

#### Heparan sulfate induces pattern formation in an ectopic blastema

**Figure 3:**
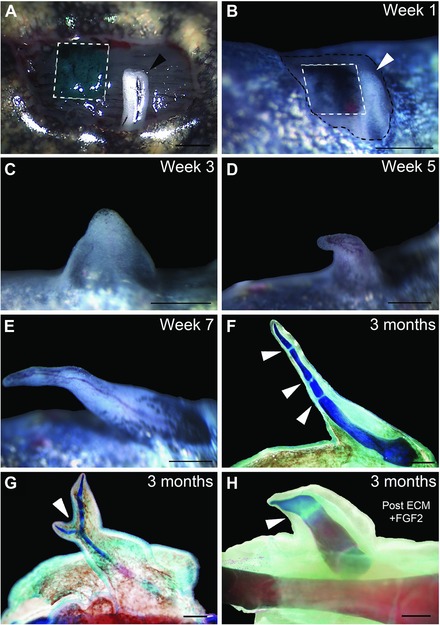
Heparan sulfate induces ectopic limb pattern during regeneration. (A) Example of the accessory limb model surgery illustrating a wound that has received an artificial extracellular matrix (ECM) graft (green, outlined with dashed white lines) and a deviated nerve (white with black arrowhead). (B) One week after nerve deviation (white with white arrowhead) and artificial ECM graft (black India ink, outlined with dashed white lines). Growth of induced blastema 3 weeks (C), 5 weeks (D) and 7 weeks (E) after surgery. (F) Whole‐mount staining of the skeletal pattern formed in (E). Arrowheads indicate joint‐like segmentations. (G) Example of a bifurcated limb pattern (indicated by white arrowhead) induced by an artificial ECM graft. (H) Example of pattern induced by an FGF2‐treated posterior ECM graft. Scale bars 1 mm.

To determine if heparan sulfate alone are able to pattern an ectopic limb, we created and grafted an artificial matrix (artificial ECM) composed of varying amounts of heparan sulfate combined with type I collagen. The artificial ECM was grafted into nerve‐deviated anterior wounds as described above for decellularized ECM (Fig. 3A).

Grafts of artificial ECM containing only type I collagen resulted in the formation of ectopic blastemas without pattern, a result that is comparable to the result when no ECM was grafted (Tables 1, 2; Fig. 2B; *P* = 1.0). The frequency of induction of ectopic blastemas was not affected by artificial ECM grafts containing medium amounts of heparan sulfate (med HS, Table 2; Fig. 2B; 19 of 21 wounds formed blastemas; *P* = 1.0). However, med‐HS artificial ECM grafts induced complex limb skeletal patterns in nearly half (8/19 = 42%) of the ectopic blastemas (Table 2; Figs 2B, 3; *P* < 0.001). The induced patterns contained multiple skeletal elements with joints (Fig. 3F, arrowheads), as well as elements that bifurcated (Fig. 3G, arrowhead). All of the ectopic patterns included distal elements that tapered with the same morphology as terminal phalanges.

**Table 2:** Heparan sulfate is sufficient to induce pattern formation in ectopic blastemas

Graft type	Total	Blastema*	Pattern**
Collagen only	6	6 (100%)	0
Med‐HS artificial ECM	21	19 (90%)	8 (42%)
Low‐HS artificial ECM	7	3 (43%)	1 (33%)
High‐HS artificial ECM	5	5 (100%)	0

*Percentage of total number of grafts that developed an ectopic blastema; **percentage of ectopic blastemas that developed skeletal elements with pattern.

The frequency of ectopic blastema formation and pattern formation was altered in response to changes in the concentration of heparan sulfate in the artificial ECM. When artificial ECM containing low amounts of heparan sulfate was grafted, the frequency of blastema formation decreased by 50% (Table 2; Fig. 2B; 43% compared to 90% for med‐HS artificial ECM; *P* = 0.02), and only one of the ectopic blastemas formed pattern. In contrast, increasing the amount of heparan sulfate in the artificial ECM had no effect on the frequency of blastema formation (*P* = 1.0); however, high‐HS artificial ECM grafts did not induce pattern formation in these blastemas (Table 2; Fig. 2B). The observed changes in bioactivity of artificial ECM containing heparan sulfate when grafted into nerve‐deviated wounds is consistent with the hypothesis that heparan sulfate has the potential to regulate the regeneration response (both blastema formation and pattern formation). These results demonstrate that heparan sulfate can induce pattern formation in an ectopic blastema.

#### The ECM can function as a source of growth factors for regulating regeneration

**Figure 4:**
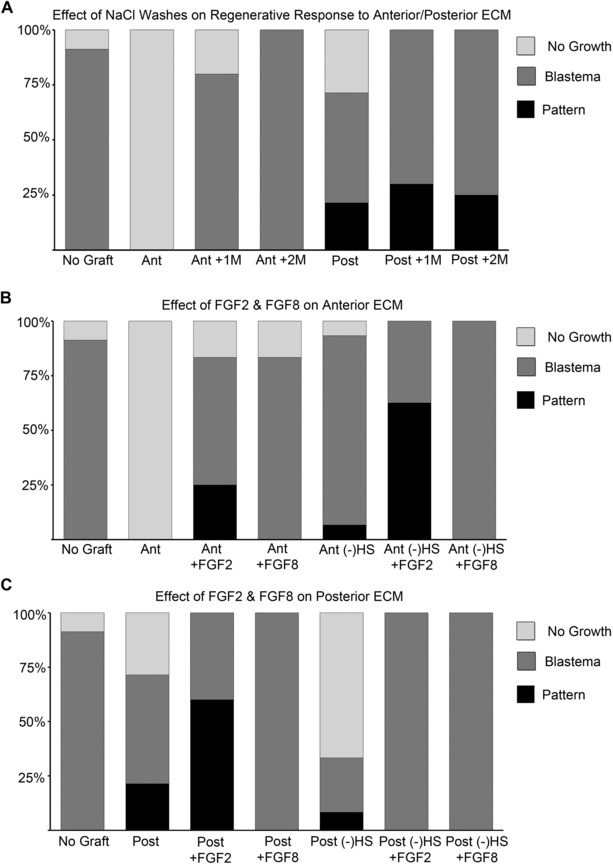
Position‐specific effects of treating extracellular matrix (ECM) grafts with NaCl, FGF2 or FGF8 on blastema and pattern formation. (A) Anterior (Ant) and posterior (Post) ECM grafts were washed with either 1 or 2 mol/L NaCl prior to grafting. (B) Anterior ECM grafts (Ant) were soaked in either FGF2 or FGF8 prior to grafting. (−)HS, HepIII treated to remove HS from the ECM. (C) Posterior ECM grafts (Post) were soaked in either FGF2 or FGF8 prior to grafting. (−)HS, HepIII treated to remove HS from the ECM.

Heparan sulfate have binding sites for growth factors that enable cell‐surface heparan sulfate proteoglycans to act as co‐receptors that are required for signaling (e.g., FGF signaling) (Sarrazin et al. 2011). Consequently, heparan sulfate associated with the ECM grafts could be affecting regeneration (blastema and pattern formation) by delivering growth factors to anterior cells in the wound. To determine whether growth factors bound to heparan sulfate affected regeneration in our assay, we washed the ECM grafts with salt (NaCl) to remove any bound factors prior to grafting. The most dramatic result was that NaCl treatment completely rescued blastema formation in wounds that received anterior ECM grafts (*P* < 0.001); however, removal of growth factors did not induce pattern formation in any of these blastemas (Table 3; Fig. 4A). In contrast, NaCl treatment did not have an effect on the ability of the ECM to affect pattern formation (i.e., posterior ECM induced pattern formation and anterior ECM did not). Based on these results, we hypothesize that there are factors bound to heparan sulfate that inhibit blastema formation in anterior wounds, but these bound factors are not active in regulating pattern formation during regeneration.

**Table 3:** Washing of anterior ECM grafts with high concentrations of NaCl rescues blastema formation but does not induce pattern formation

Graft type	Total	Blastema*	Pattern**
Anterior ECM	8	0 (0%)	0
Anterior ECM + 1 mol/L NaCl	10	8 (80%)	0
Anterior ECM + 2 mol/L NaCl	8	8 (100%)	0
Posterior ECM	14	10 (71%)	3 (30%)
Posterior ECM + 1 mol/L NaCl	10	10 (100%)	3 (30%)
Posterior ECM + 2 mol/L NaCl	8	8 (100%)	2 (25%)

*Percentage of total number of grafts that developed an ectopic blastema; **percentage of ectopic blastemas that developed skeletal elements with pattern.

#### Position‐specific mediation of FGF signaling regulates blastema formation and pattern formation

Heparan sulfate plays a critical role in the regulation of FGF signaling (Rapraeger et al. 1991; Yayon et al. 1991; Olwin & Rapraeger 1992) and both FGF2 and FGF8 are expressed and function during limb regeneration (Boilly et al. 1991; Mullen et al. 1996; Christen & Slack 1997; Christensen et al. 2001; Han et al. 2001). Additionally there may be distinct heparan sulfate requirements to mediate specific FGF signaling (Guimond et al. 1993). We therefore hypothesized that differential regulation of FGF2 and FGF8 signaling by anterior or posterior heparan sulfate could be functionally involved in the experimental results observed in response to ECM grafting. We therefore soaked ECM preparations (with or without prior treatment with HepIII) in either FGF2 or FGF8 prior to grafting into anterior wounds.

Pretreatment of anterior ECM grafts with either FGF2 or FGF8 rescued blastema formation (Table 4; Fig. 4B; *P* < 0.001). As discussed above, NaCl treatment of anterior ECM also rescued blastema formation, presumably by removing inhibitory factors. It is therefore possible that both FGF2 and FGF8 competed with inhibitory factors for binding to the ECM, and were able to rescue blastema formation by displacing those factors. In addition to the ability to rescue blastema formation, FGF2 treatment also induced pattern formation in 30% of the grafts (Table 4; Fig. 4B; *P* < 0.001). The ability of FGF2 to induce pattern formation was even more evident in anterior ECM pretreated with HepIII to remove heparan sulfate (62% compared to 30% without HepIII treatment). Treatment of anterior ECM with FGF8 did not induce any pattern formation whether or not the grafts had been treated with HepIII. These results suggest that anterior ECM mediated a differential regeneration response whether pretreated with FGF2 or FGF8 (*P* < 0.01).

**Table 4:** Effects of FGF2 and FGF8 on blastema and pattern formation in response to anterior ECM grafts

Graft type	Total	Blastema*	Pattern**
Anterior ECM	8	0 (0%)	0
Anterior ECM + FGF2	12	10 (83%)	3 (30%)
Anterior ECM + FGF8	6	5 (83%)	0
Heparin lyase III anterior ECM	15	14 (93%)	1 (7%)
Heparin lyase III anterior ECM + FGF2	8	8 (100%)	5 (62%)
Heparin lyase III anterior ECM + FGF8	11	11 (100%)	0

*Percentage of total number of grafts that developed an ectopic blastema; **percentage of ectopic blastemas that developed skeletal elements with pattern.

Although untreated posterior ECM grafts in general did not inhibit blastema formation, the frequency of ectopic blastema formation increased in response to treatment with either FGF2 or FGF8 (from 70% to 100%; Table 5; Fig. 4C). This was the same result observed with NaCl treatment (Table 3; Fig. 4A), suggesting that FGF2 and FGF8 compete with inhibitory factors for binding to both anterior and posterior ECM, and are able to rescue/increase the frequency of blastema formation by displacing those inhibitory factors. As with anterior ECM grafts, FGF2 treatment but not FGF8 treatment increased the frequency of pattern formation (60% compared to 30% for untreated posterior ECM grafts; *P* = 0.03). The induced pattern in response to FGF2 treatment (Fig. 3H) was comparable to that observed for untreated posterior ECM grafts. Strikingly, treatment with FGF8 completely inhibited the ability of posterior ECM to induce pattern formation. In contrast to anterior ECM, the ability of FGF2‐treated posterior ECM to induce pattern was dependent on heparan sulfate such that HepIII pretreatment inhibited pattern formation (Table 5; Fig. 4C). Taken together these results further demonstrate the differential regenerative response between anterior and posterior ECM in terms of FGF signaling.

**Table 5:** Effects of FGF2 and FGF8 on blastema and pattern formation in response to posterior ECM grafts

Graft type	Total	Blastema*	Pattern**
Posterior ECM	14	10 (71%)	3 (30%)
Posterior ECM + FGF2	10	10 (100%)	6 (60%)
Posterior ECM + FGF8	6	6 (100%)	0
Heparin lyase III posterior ECM	12	4 (33%)	1 (25%)
Heparin lyase III posterior ECM + FGF2	7	7 (100%)	0
Heparin lyase III posterior ECM + FGF8	11	11 (100%)	0

*Percentage of total number of grafts that developed an ectopic blastema; **percentage of ectopic blastemas that developed skeletal elements with pattern.

There are at least three intriguing and consistent conclusions that emerge from these experiments. First, treatment with either FGF2 or FGF8 increased the frequency of blastema formation, suggesting that there is an inhibitor of blastema formation that is displaced by FGFs (as well as by NaCl). Second, FGF2 increased the frequency of pattern formation, whereas FGF8 inhibited pattern formation. Finally, posterior but not anterior ECM‐mediated pattern induction was dependent on heparan sulfate, as depletion of heparan sulfate inhibited pattern formation induced by FGF2‐treated posterior ECM, but doubled pattern formation by anterior ECM. Together, these results are consistent with the hypothesis that FGF signaling plays critical roles in blastema formation and pattern formation, and that there are mechanisms for regulating position‐specific FGF signaling during regeneration.

#### Heparan sulfate sulfotransferases are differentially expressed in anterior and posterior blastema cells during regeneration

Based on the results above, it appears that heparan sulfate‐mediated growth factor signaling is different between the anterior and posterior ECM, and is associated with the positional interactions that control blastema formation and pattern formation during regeneration. Presumably these differences arose during limb development, and they are reestablished during limb regeneration. We thus hypothesized that there must be either quantitative differences in the levels of different heparan sulfate, and/or qualitative differences in the types of sulfation patterns (Yayon et al. 1991). To date we have not been able to detect significant differences in heparan sulfate composition using HPLC‐MS (data not shown). However, we have identified the predicted differences in the expression of heparan sulfate sulfotransferases that would function to establish differential sulfation patterns.

**Figure 5:**
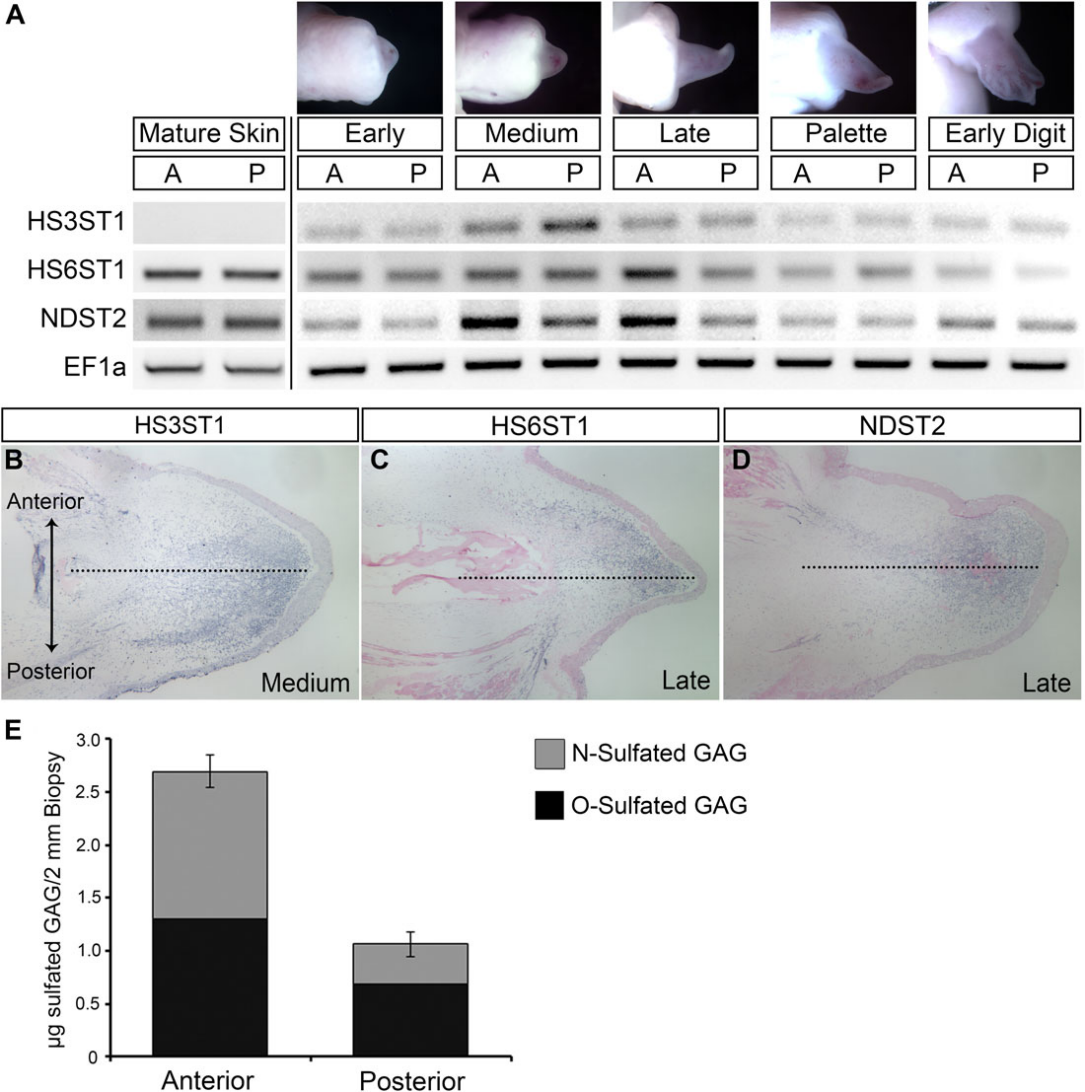
Anterior/posterior differential expression of heparan sulfate sulfotransferases during limb regeneration. (A) RT‐PCR expression analysis of the heparan sulfate sulfotransferases HS3ST1, HS6ST1, and NDST2 in the anterior and posterior of blastema at progressive stages of limb regeneration. EF1α was used as a normalizing expression control. A (anterior) and P (posterior) blastema portions from early bud, medium bud, late bud, palette, and early digit stages of blastema growth. (B), (C), (D) RNA *in situ* hybridization for expression of HS3ST1 (B), HS6ST1 (C), and NDST2 (D). Dotted lines indicate midline between anterior (top) and posterior (bottom) sides of the limb that correspond to the plane of bisection for collection of the blastema tissue samples. (E) Higher levels of GAG sulfation in anterior axolotl skin as measured by the Blyscan sulfated GAG assay. Error bars represent the standard error of the mean from three independent biological samples. Statistically significant difference between anterior and posterior sulfated GAG levels, *P* < 0.001.

Our search of the Ambystoma EST database (http://www.ambystoma.org/genome‐resources/5‐gene‐and‐est‐database) identified 10 axolotl heparan sulfate sulfotransferases expressed in axolotl tissues (Table 6). We used semi‐quantitative RT‐PCR to determine which of these were expressed in either uninjured or regenerating limb tissues. Of the six genes that were expressed during limb regeneration, three (heparan sulfate 3‐*O*‐sulfotransferase 1 [HS3ST1], heparan sulfate 6‐*O*‐sulfotransferase 1 [HS6ST1], and *N*‐deacetylase/*N*‐sulfotransferase 2 [NDST2]) were expressed differentially between anterior and posterior regions of regenerating limb blastemas (Table 6; Fig. 5). Expression of two of these (HS6ST1 and NDST2) was detected in uninjured limb skin. For all three, expression appeared to increase coincident with blastema formation and growth, with maximal levels observed around the medium to late bud stages of regeneration (Fig. 5A). At that stage, expression of NDST2 and HS6ST1 appeared to be higher in the, anterior half of the blastema, whereas expression of HS3ST1 appeared to be higher in the posterior half.

**Table 6:** Expression of heparan sulfate sulfotransferases identified from the Ambystoma EST database

Gene	Uninjured skin	Blastema
NDST1	+	+ (A = P)
NDST2	+	+ (A > P)
NDST3	−	−
NDST4	−	−
HS6ST1	+	+ (A > P)
HS2ST1	+	+ (A = P)
HS3ST1	−	+ (P > A)
HS3ST2	+	−
HS3ST3B1	−	+ (A = P)
HS3ST4	−	−

+, indicates the presence of a band on RT‐PCR; −, indicates that no band was observed; A, indicates the level of expression by anterior blastema cells; P, indicates the level of expression by posterior blastema cells.

We confirmed that these three genes were differentially expressed along the anterior−posterior axis of medium−late bud blastemas by RNA *in situ* hybridization (Fig. 5B−D). Expression of all three genes was detected in blastema mesenchyme cells in both anterior and posterior regions. Consistent with the RT‐PCR results, there was a gradient of expression for each, with the domain of expression for HS3ST1 being more limited in the anterior compared to the posterior, and the posterior domain being more limited for HS6ST1 and NDST2.

The observed anterior/posterior differential expression of heparan sulfate sulfotransferases was consistent with the hypothesis that there would be quantitative and qualitative differences in heparan sulfate sulfation between anterior and posterior limb skin. To measure relative levels of sulfated GAGs in axolotl anterior and posterior limb skin, we used the dimethyl‐methylene blue dye binding Blyscan assay. We found that there was 2.5× (*P* < 0.01) more sulfated GAGs in the anterior skin, and that there was a higher proportion of *N*‐sulfated GAGs in the anterior skin (Fig. 5E). Both observations were consistent with the heparan sulfate sulfotransferase expression data (higher levels of NDST2 expression in the anterior region of the blastema). Taken together, these data are consistent with the hypothesis that different compositions of heparan sulfate between anterior and posterior limb skin are achieved by different expressions of heparan sulfate sulfotransferase genes.

#### Mouse limb ECM induces heparan sulfate‐dependent, position‐specific and developmental stage‐dependent pattern formation in axolotl blastemas

Axolotls have an amazing ability to regenerate after traumatic injury; however, this ability does not seem to be conserved in mammals. One strategy for enhancing the regenerative capacity of humans is to identify the requirements for successful regeneration in axolotls (Endo et al. 2004; Muneoka et al. 2008), which would allow for the identification of the necessary steps that do not occur following injury in mammals. The ability to assay signaling by the ECM rather than cells allowed us to expand our study to include mammals. This has not been possible previously because axolotl and mouse cells cannot survive and/or function normally at the same temperature (37°C versus 20°C).

**Figure 6:**
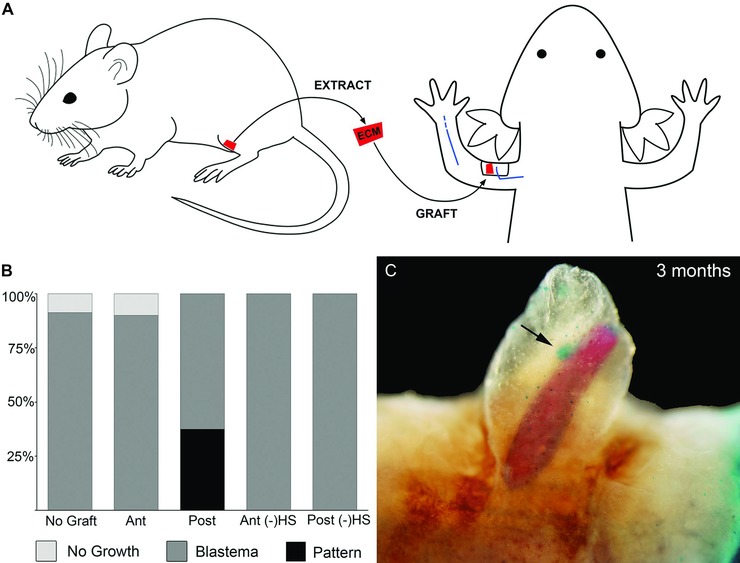
Posterior mouse extracellular matrix (ECM) induces pattern formation by axolotl blastema cells. (A) Schematic diagram of surgical procedures. Mouse hindlimb (femur) skin samples (red) were collected from mice for ECM extraction followed by grafting into an axolotl skin wound with a deviated brachial nerve (blue). (B) Differential regenerative responses to grafts of mouse‐derived anterior or posterior ECM from skin samples at postnatal day 3−4 either with or without heparin lyase III treatment. Ant, anterior; Post, posterior; (−)HS, heparin lyase III treatment to remove heparan sulfate from the ECM. (C) Whole‐mount skeletal preparation of a skeletal‐like element formed by axolotl blastema cells in response to an ECM graft from the posterior of a mouse limb at about 2 months post‐grafting: red, ossified bone; blue, cartilage indicated by arrow.

**Figure 7:**
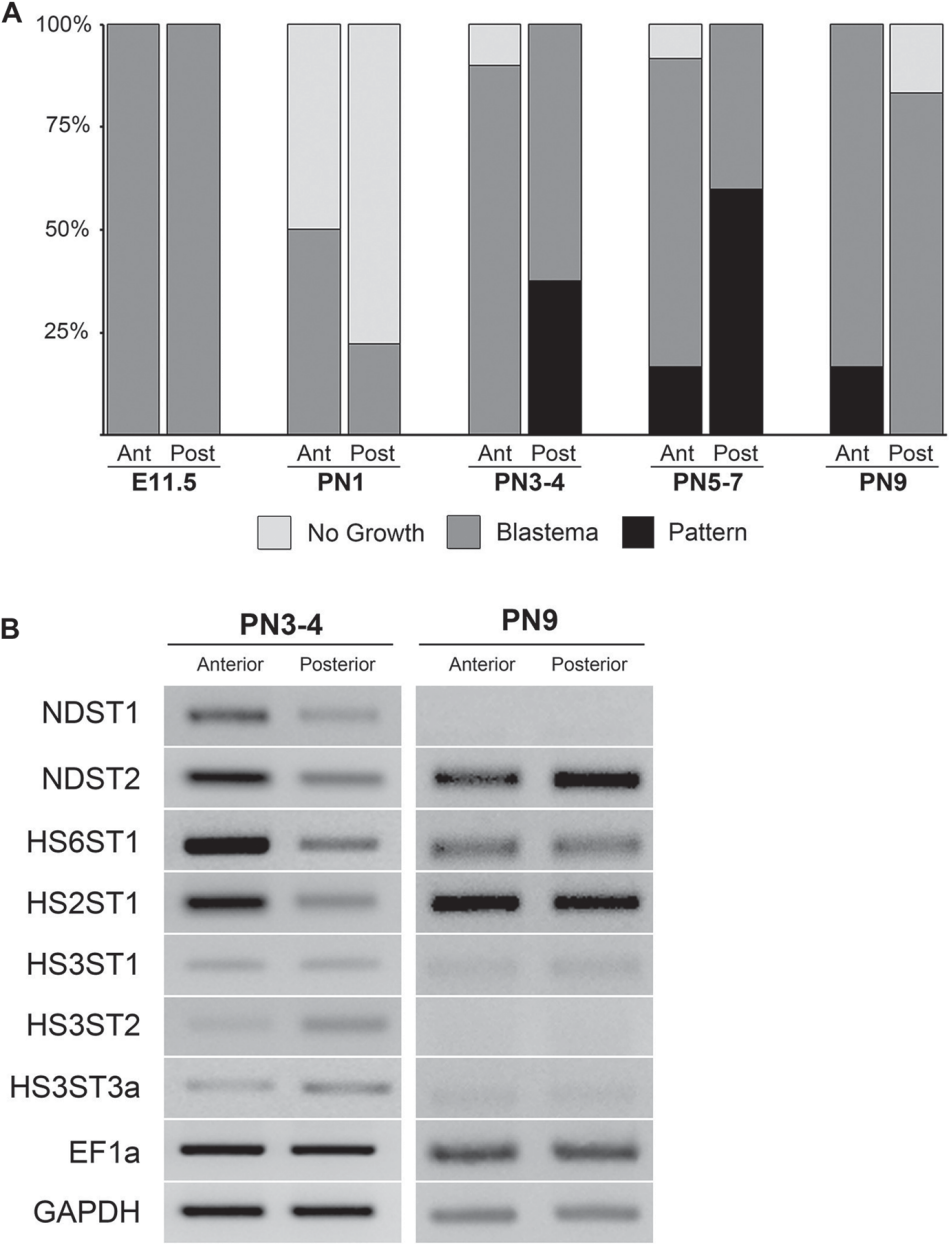
Characterization of mouse limb skin effects on the induction of ectopic limb skeletal elements in axolotl blastemas, and the expression of sulfotransferases. (A) Age‐dependent position‐specific ability of mouse‐derived extracellular matrix grafts to induce axolotl blastema cells to form ectopic limb skeletal elements. E11.5, mouse embryonic day 11.5; PN1, mouse postnatal day 1; PN3−4, mouse postnatal day 3−4; PN5−7, mouse postnatal day 5−7; PN9, mouse postnatal day 9; Ant, anterior; Post, posterior; No Growth, no growth observed (example Fig. 1G); Blastema, growth of blastema observed (example Fig. 1H) but no pattern (no skeletal elements induced); Pattern, blastema growth followed by pattern formation confirmed by Alcian Blue and Alizarin Red skeletal preparation (example Fig. 6C). (B) Age‐specific anterior/posterior differential expression of heparan sulfate sulfotransferases in mouse skin assayed by RT‐PCR. Expression of the sulfotransferases was analyzed in the anterior and posterior skin of mouse hindlimb (femur) at PN3−4 and PN9. As observed for axolotl skin, N, 6‐*O*‐ and 2‐*O*‐sulfotransfereases were more highly expressed in the anterior of the limb in PN3−4 (compare to Fig. 5). Differential expression was diminished by PN9. EF1α and GAPDH were used as loading controls.

To test whether mouse limb ECM has specific signaling abilities that are comparable to axolotl limb ECM, we repeated the experiment of grafting urea‐extracted/HepIII‐treated anterior and posterior ECM using prenatal/neonatal mouse limb skin rather than axolotl limb skin (Fig. 6A). Mouse limb ECM grafts collected from mice between E11.5 and PN9 induced a range of regenerative responses when grafted into nerve‐deviated axolotl wounds (Figs 6B, C, 7A; Tables 7, 8). Nerve‐deviated wounds almost always form an ectopic blastema (Endo et al. 2004; Satoh et al. 2007). When ECM collected from PN1 mouse hindlimbs was grafted in a nerve‐deviated axolotl wound, ectopic blastemas often did not form (Fig. 7A, Table 8; 70% *N* = 24; *P* < 0.0001). Since a nerve had been deviated surgically, the expectation was that all wounds should have formed an ectopic blastema (Endo et al. 2004; Satoh et al. 2007). However, PN1 mouse limb ECM grafts inhibited axolotl blastema formation at a high frequency. This inhibitory activity appeared higher in ECM derived from the posterior side of the mouse limb (78% compared to 50% for anterior‐derived grafts, Fig. 7A, Table 8). In contrast to PN1 ECM grafts, ECM grafts collected from limb buds of E11.5 mice had no effect on blastema formation, and all limbs formed an ectopic blastema as expected (Fig. 7A, Table 8). Grafts of ECM from PN3−4, PN5−7 and PN9 were evident at subsequent time points; however, none of the E11.5 or PN1 grafts persisted beyond 1 week post‐grafting (not illustrated). Taken together, these data indicate that there are ontogenetic changes in mouse ECM that regulate its ability to signal to axolotl cells either to inhibit blastema formation or to induce ectopic limb pattern, as well as to make it more or less resistant to remodeling and removal by the axolotl wound environment. Although the grafted mouse ECM appears to have changed, the axolotl host cells remain the same. Thus, further studies of the interaction between mouse ECM and axolotl connective tissue cells in an axolotl wound environment could lead to novel insights into the regulation of fibroblast biology and fibrosis. The ability of the axolotl wound microenvironment to resorb the E11.5 and PN1 ECM grafts may be related to the observation that axolotl skin wounds heal without scar formation (Endo et al. 2004; Murawala et al. 2012; Denis et al. 2013).

**Table 7:** The position‐specific ability of anterior/posterior mouse limb skin ECM grafts to induce pattern formation in ectopic axolotl blastemas is dependent on heparan sulfates

Graft type	Total	Blastema*	Pattern**
No graft	23	21 (91%)	0
PN3−4 mouse anterior ECM	10	9 (90%)	0
PN3−4 mouse posterior ECM	8	8 (100%)	3 (37%)
Heparin lyase III PN3−4 mouse anterior ECM	8	8 (100%)	0
Heparin lyase III PN3−4 mouse posterior ECM	10	10 (100%)	0

*Percentage of total number of grafts that developed an ectopic blastema; **percentage of ectopic blastemas that developed skeletal elements with pattern

**Table 8:** The position‐specific ability of anterior/posterior mouse limb skin ECM grafts to induce pattern formation in ectopic axolotl blastemas is dependent on the developmental stage of the donor mouse

Graft type	Total	Blastema*	Pattern**
E11.5 mouse anterior ECM	8	8 (100%)	0
E11.5 mouse posterior ECM	8	8 (100%)	0
PN1 mouse anterior ECM	6	3 (50%)	0
PN1 mouse posterior ECM	18	4 (22%)	0
PN3‐4 mouse anterior ECM	10	9 (90%)	0
PN3‐4 mouse posterior ECM	8	8 (100%)	3 (37%)
PN5‐7 mouse anterior ECM	12	11 (92%)	2 (18%)
PN5‐7 mouse posterior ECM	5	5 (100%)	3 (60%)
PN9 mouse anterior ECM	6	6 (100%)	1 (17%)
PN9 mouse posterior ECM	6	5 (83%)	0

*Percentage of total number of grafts that developed an ectopic blastema; **percentage of ectopic blastemas that developed skeletal elements with pattern.

Mouse skin ECM grafts from later stages (PN3−PN9) induced axolotl blastema cells to form skeletal elements (Fig. 6C, Table 8). The structures induced by mouse posterior ECM were elongated, but unlike the pattern induced by axolotl ECM they did not form joints and did not taper distally. These skeletal structures also stained with Alizarin Red, which stains calcified tissues (bone and calcified cartilage), in contrast to the cartilaginous elements (stained with Alcian Blue) induced by axolotl ECM grafts (Figs 1, 3). In addition to the well‐formed Alizarin Red positive elements, small nodules of Alcian Blue positive cartilage were observed (arrow, Fig. 6C). Thus posterior mouse ECM was able to induce skeletal pattern formation by axolotl cells, but these cells differentiated into calcified tissue that would be the fate of mouse mesenchymal cells, rather than into cartilage that would be their normal axolotl fate.

The ability to induce limb pattern varied between anterior and posterior mouse ECM grafts such that PN3−4 posterior ECM induced pattern (38%, *N* = 8) but PN3−4 anterior ECM did not (0%, *N* = 9; *P* = 0.07). The frequency of skeletal pattern induction increased for PN5−7 posterior ECM grafts (60%, *N* = 5), but was lost by PN9 (Fig. 7, Table 8). A low level of skeletal pattern induction was observed for anterior ECM grafts from PN5−7 (18%, *N* = 11) as well as from PN9 (17%, *N* = 6). As with grafts of axolotl limb ECM, this signaling ability was dependent on the presence of heparan sulfate since it was lost when the grafts were treated with heparitinase (HepIII) (Fig. 6B, Table 7). Thus the ability of mouse limb skin ECM to affect ectopic axolotl limb regeneration was position specific, developmental stage dependent, and heparan sulfate proteoglycan dependent.

#### The expression of heparan sulfate sulfotransferases is differentially regulated in both time and space

Based on the finding that the ability of mouse limb skin ECM to induce pattern formation in axolotl blastemas is dependent on heparan sulfate, we hypothesized that there is differential regulation of heparan sulfate across the anterior and posterior axis established by differential expression of heparan sulfate sulfotransferases during mouse skin development, as we had discovered for axolotl limb skin.

We analyzed the expression of seven mouse heparan sulfate sulfotransferases that were expressed in mouse hindlimb skin in PN3−4 and PN9 stage mice by semi‐quantitative RT‐PCR (Fig. 7B). Similar to axolotl heparan sulfate sulfotransferase expression during regeneration (Fig. 5A, B), NDST2 and HS6ST1 were more highly expressed in the anterior mouse limb skin at PN3−4. In addition, NDST1 and HS2ST1 were more highly expressed in PN3−4 anterior mouse limb skin (Fig. 7B). Although expression of HS3ST1 was not higher in the posterior skin as observed in the axolotl, expression of both HS3ST2 and HS3ST3a appeared to be higher in posterior skin. By PN9, expression of NDST1, HS3ST1, HS3ST2, and HS3ST3a were no longer expressed at detectable levels, and NDST2 appeared to change to a more posterior expression in mouse limbs (Fig. 7B). Taken together the mouse heparan sulfate sulfotransferase expression patterns correlated well with the position‐specific age‐dependent ability of mouse limb skin ECM to induce limb pattern formation.

### Discussion

The ability to regenerate a limb is dependent on both regeneration‐competent cells and the information that regulates the behavior of those cells in order to reform the structure and reestablish the function of the amputated limb (Muneoka et al. 2008). The necessity of having positional information is evident from studies involving the formation of limbs de novo on the side of the arm of axolotls (Endo et al. 2004; Satoh et al. 2007). Nerve‐associated signaling is necessary to recruit regeneration‐competent cells to form an ectopic blastema, but is not sufficient to induce regeneration of a limb. Limb formation requires the presence of information both from the wound site and from the opposite side of the limb (e.g., by grafting of posterior skin to an anterior wound). Regeneration‐competent cells are recruited from the stump either by dedifferentiation (e.g., connective tissue cells) or by activation of adult stem cells (e.g., satellite cells that give rise to muscle) to form the blastema (Bryant et al. 2002; Sandoval‐Guzmán et al. 2014). The molecular nature of the positional information required for regeneration is largely unknown, although it is localized to loose connective tissues (Bryant et al. 2002; Nacu et al. 2013). Our findings indicate that the molecular basis of positional information involves interactions between ECM with position‐specific heparan sulfate modification patterns, growth factors (FGFs in particular), and regeneration‐competent cells. We have demonstrated that heparan sulfate can induce de novo limb pattern formation during regeneration. We have also discovered that the role of heparan sulfate in mediating positional information is conserved in mammals.

Pattern formation by anterior blastema cells in response to posterior ECM grafts is predicted by the polar coordinate model (French et al. 1976; Bryant et al. 1981), and is comparable to the response of these same cells to posterior skin grafts in the ALM (Endo et al. 2004; Satoh et al. 2007). In both cases, the interpretation of the data is that cells respond to differences in positional information by proliferating and making new pattern. The formation of ectopic limb pattern in the present study is consistent with the hypothesis that cell‐free posterior ECM encodes positional information that is different from that of anterior cells. The amount of ectopic limb pattern induced in response to posterior ECM grafts (this study) is less than in response to posterior skin grafts that contain both cells and ECM (Endo et al. 2004). Since the ECM is synthesized by the cells, the matrix‐encoded positional information is dependent on the presence of cells with the same positional information. Therefore, it is likely that positional signaling by posterior ECM is sufficient to induce pattern formation but is not sustained over a long enough period of time to allow for the complete limb pattern to form. Grafted limbs were allowed to develop until the induced outgrowth no longer increased in size or formed more complex pattern when observed over three consecutive observations, typically over a 3‐month period. Therefore, it is unlikely that more pattern would have formed if these limbs were collected at a later time point.

The nerve‐induced ectopic blastemas were formed by recruitment of dermal cells surrounding the margin of the wound (Endo et al. 2004), and thus we presume these are the cells that form the ectopic skeletal elements. In our experiments, we induced ectopic blastemas on the anterior side of the arm, and therefore cells derived from progenitors with only anterior positional information made the new skeletal elements. Thus some of those cells presumably were reprogrammed by interactions with the grafted posterior ECM in order to acquire a more posterior identity. Differentiated stump tissues can reprogram the positional identity of dedifferentiated blastema cells (McCusker & Gardiner 2013), and it appears that cell‐free ECM from uninjured skin can do this as well. The ability of ECM grafts treated with FGF2 to induce ectopic limb patterns indicates that dedifferentiated anterior blastema cells can be reprogrammed to acquire a more posterior identity by regulating FGF signaling. Thus increasing FGF2 signaling induces (Table 4; Fig. 4B) or enhances (Table 5; Fig. 4C) pattern formation. Future studies to optimize and enhance the signals involved in time and space presumably will lead to increasing the complexity of pattern formation in the anterior−posterior axis in addition to what we have observed already along the proximal−distal axis.

**Figure 8:**
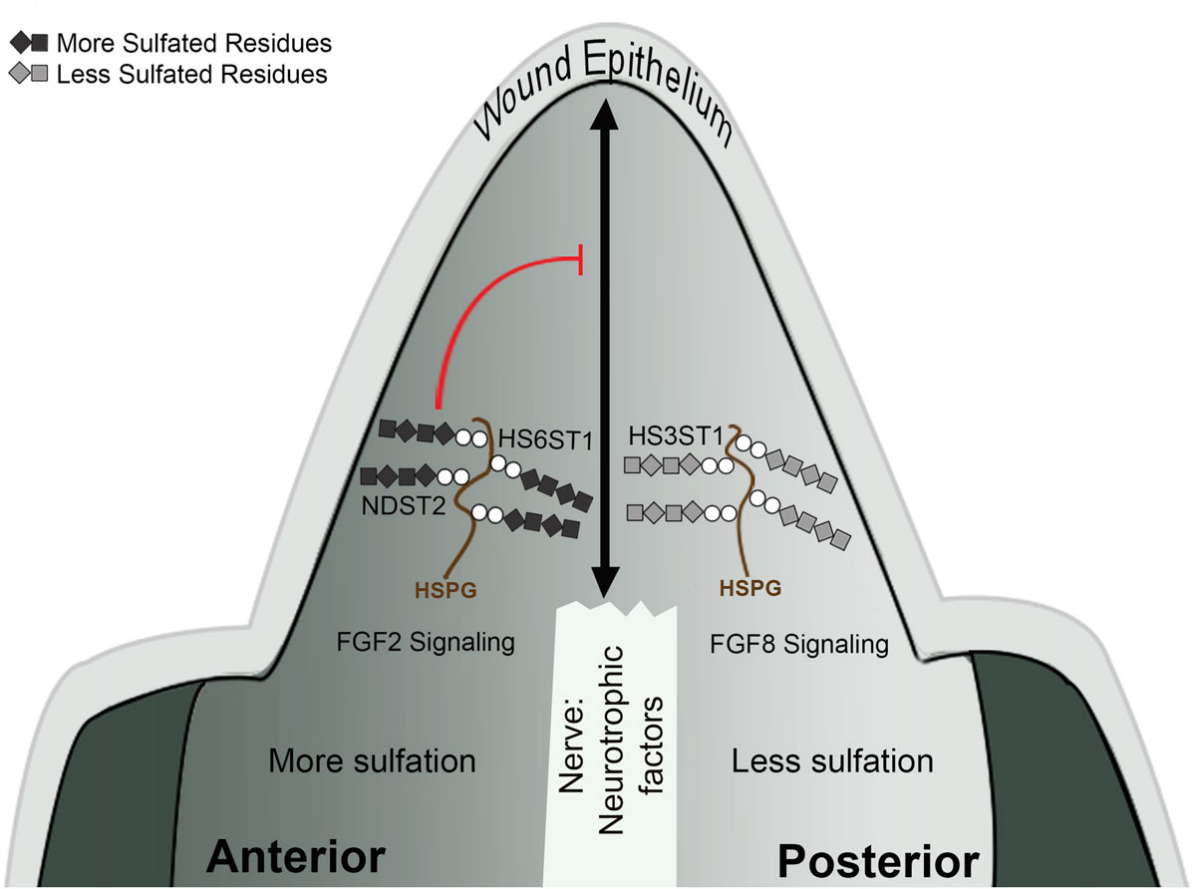
Model of heparan sulfate regulation of axolotl limb regeneration. In the regenerating limb blastema, the nerve interacts with the wound epithelium to release neurotrophic factors that induce blastema formation and generate regeneration‐competent cells. Positional information from the limb stump (or supplied by extracellular matrix [ECM] grafts) then induces pattern formation. Differential expression of HS3ST1, HS6ST1, and NDST2 results in qualitative and quantitative differences of heparan sulfate sulfation across the anterior−posterior axis. Differentially sulfated heparan sulfate proteoglycans (HSPG) in the anterior or posterior ECM regulate FGF2 and FGF8 signaling preferentially to reestablish the anterior−posterior limb axis in the blastema. Grafted heparan sulfate in anterior‐derived ECM can inhibit nerve‐induced blastema formation (reference lines).

Based on our findings, we hypothesize that sulfation patterns of the ECM are modified in both time and space, resulting in both qualitative and quantitative regulation of blastema formation and pattern formation (Fig. 8). By this view, these modifications of heparan sulfate function in part by their ability to differentially regulate FGF signaling, particularly with regard to controlling pattern formation along the anterior−posterior limb axis. A role for FGF signaling in both the initiation of blastema formation and the maintenance of blastema growth has been hypothesized previously. In addition, there are multiple sources of FGFs in the blastema, including the nerve, apical epithelium, and apical blastema mesenchyme cells (Boilly et al. 1991; Mullen et al. 1996; Christen & Slack 1997; Christensen et al. 2001; Han et al. 2001; Satoh et al. 2008; Sarrazin et al. 2011). Thus the necessary signals (FGFs) are present at the appropriate time and places in order to be regulated by specific heparan sulfate proteoglycans in the ECM. This model is consistent with both the ECM grafting and gene expression data.

Our ECM grafting data also indicate the presence of a regeneration inhibitory activity that is associated with anterior ECM, but not with posterior ECM. Since this activity can be removed by washing with high concentrations of NaCl, it presumably is a factor that binds to the ECM. This anti‐regeneration activity is also removed by treatment with HepIII, and thus most likely is a factor that is bound by anterior‐specific heparan sulfate modifications. In addition, regeneration can be rescued by treating anterior ECM with FGF2, which presumably out‐competes the inhibitory factor for binding to anterior heparan sulfate. The lack of pattern formation by treatment with FGF8 is further evidence for the qualitative specificity of the ECM modifications involved in regulating this phenomenon. Different sulfation modifications of heparan sulfate are known to specifically regulate the affinity for different members of the FGF family (Guimond et al. 1993). Since we did not observe anti‐regeneration activity associated with posterior ECM, we presume that there are posterior‐specific heparan sulfate modifications that do not bind the inhibitory factor, or that synthesis of the factor is restricted to the anterior portion of the limb. Further studies of position‐specific sulfation patterns of heparan sulfate will be required to answer these questions.

Evidence that quantitative changes in heparan sulfate can regulate the regenerative response comes from the responses of wounds to artificial ECM grafts (Table 2; Figs 2B, 3). At an intermediate concentration of heparan sulfate, blastemas form and nearly half of them form complex patterns. At lower concentrations of heparan sulfate, blastema formation is inhibited, whereas at higher concentrations pattern formation is inhibited. The spatial expression patterns of the heparan sulfate sulfotransferases predict that there are differences in the levels of sulfation in anterior ECM compared to posterior ECM. Both HS6ST1 and NDST2 were expressed at higher levels in the anterior portion of blastemas, and both add high levels of sulfate to GAG substrates (Thacker et al. 2014). In contrast, HS3ST1 was expressed at higher levels in the posterior portion of blastemas, but it has a low level of sulfation activity (Thacker et al. 2014). These data predict that the overall level of ECM sulfation is higher on the anterior side of the blastema compared to the posterior side, which was verified by the Blyscan assay (Fig. 5E).

For both the mouse and the axolotl, the underlying mechanism for ECM‐mediated signaling is associated with position‐specific differential expression of heparan sulfate sulfotransferases. A similar model of regulation of growth and pattern formation by spatial and temporal modifications of heparan sulfate has been hypothesized for developing chick limb buds (Nogami et al. 2004; Kobayashi et al. 2010). HS6ST1 was identified as being expressed at higher levels in the anterior of the developing chick limb bud, similar to our findings in axolotl limb blastemas as well as PN3−4 mouse limb skin. Knockdown of this gene resulted in altered growth and pattern formation (Kobayashi et al. 2010), suggesting that there is a conserved function for heparan sulfate modifications in both developing and regenerating limbs. In our study, heparan sulfate sulfotransferase expression in mouse limb skin correlated with the grafting results. At the developmental stages when there were anterior/posterior grafting differences (PN3−4), heparan sulfate sulfotransferases were differentially expressed. When there were fewer anterior/posterior graft differences (PN9), there was less differential heparan sulfate sulfotransferase expression. The change in mouse expression over time complements previous studies that have found that most differences between human fetal and adult skin were at the level of dermal ECM molecule expression and that dermal ECM components are important in fetal scarless wound healing (Coolen et al. 2010).

In addition to sulfotransferases, enzymes that remove sulfates (sulfatases) are also involved in modifying heparan‐sulfate‐mediated growth factor signaling. Of relevance to our findings, sulf1 and sulf2 have distinct patterns of expression during *Xenopus* limb development and regeneration (Wang & Beck 2015), and sulf2 is expressed at higher levels in the anterior region of hindlimb blastemas (Wang & Beck 2015). Collectively, these findings suggest that there is a conserved function for heparan sulfate modifications that are dynamically regulated in mouse, chick, *Xenopus*, and axolotl ECM.

The use of the ALM to assay for pro‐ and anti‐regenerative activity by the ECM has allowed us to address the critical question of whether what happens in an axolotl has relevance to what happens in mammals. We have discovered that mouse limb ECM can induce pattern formation in a heparan‐sulfate‐dependent, position‐specific manner (Table 7; Fig. 6), thus establishing a critical functional link between the response to injury in axolotls and in mice. Our data demonstrating the presence of position‐specific signaling properties of mouse limb skin are consistent with earlier studies from developing mouse limb buds in which cultured posterior mouse limb bud cells synthesized ECM that induced ectopic limb pattern when grafted into the anterior region of chick limb buds (Schaller & Muneoka 2001). The earlier finding that grafting an artificial ECM containing heparan sulfate also induced ectopic limb pattern when grafted into a chick limb bud (Schaller & Muneoka 2001) motivated us to do a comparable experiment using our assay for limb regeneration. Our finding that mouse ECM and purified ECM macromolecules (porcine heparan sulfate) can induce axolotl blastema cells to form limb structures de novo, as well as the report that mammalian growth factors (FGF and BMP) can induce blastema formation (Makanae et al. 2013), indicate that the signals regulating regeneration are conserved between salamanders and mammals. The ability of axolotl regeneration‐competent cells to respond to mouse ECM by generating mammalian‐specific ossified tissue suggests that the axolotl (ALM in particular) can be a powerful assay for mammalian positional information and differentiation signals crucial for the development of regenerative therapies.

Although many aspects of blastema formation and pattern formation appear conserved for mouse and axolotl, there are notable differences. Presumably the spatial and temporal regulation of genes associated with pattern formation (e.g., Meis, Hox, and Shh) are conserved for ECM‐induced ectopic limb formation as they are for limb development, amputation‐induced regeneration and ectopic limb formation in the ALM (Satoh et al. 2007). Nevertheless, there are differences in terms of what tissues eventually differentiate (i.e., cartilage in response to axolotl ECM versus bone in response to mouse ECM). Thus there appears to be information in the ECM that encodes information upstream of tissue‐specific differentiation. A detailed understanding of the mechanisms by which specific ECM modifications control pattern formation and differentiation (e.g., how different Hox codes might produce qualitative and/or quantitative differences in the pattern of sulfation) will require further studies. We feel that the current study is important in that it establishes ECM sulfation patterns as important in positional information. The ability of axolotl regeneration‐competent cells to respond to mouse ECM by generating mammalian‐specific ossified tissue suggests that the axolotl (ALM in particular) can be a powerful assay for mammalian positional information and differentiation signals crucial for the development of regenerative therapies.

Finally, we consider the discovery that anterior ECM can inhibit regeneration to be important because there are very few ways to inhibit salamander limb regeneration reversibly. Severing the nerves innervating the limb prevents regeneration, and if the nerves are allowed to regenerate the limb will regenerate when reinjured (Salley & Tassava 1981), indicating that nerves are necessary for regeneration. In contrast, the response to anterior ECM grafts indicates that anti‐regeneration signals are present, and that regeneration can be rescued when they are removed (e.g., by HepIII, NaCl, or FGF2 treatment). These data also point out that the lack of a regenerative response does not mean that an animal does not have the ability to regenerate. Therefore, the lack of a regenerative response (e.g., in humans) could be a consequence of inhibition rather than a lack of ability to regenerate. Since the regenerative failure in the axolotl is comparable to the response in humans, understanding how to de‐repress the inhibitory activity will allow for testing of whether regenerative failure in humans is a consequence of anti‐regenerative signaling activity. Understanding the signals and responses of cells, whether they are pro‐ or anti‐regenerative, will be critical to achieving the goal of regenerative engineering to re‐form complex structure and restore function in humans (Laurencin & Khan 2013).

## Materials and Methods

### Ethics statement

This study was carried out in accordance with the recommendations in the Guide for Care and Use of Laboratory Animals of the National Institutes of Health. The experimental work was conducted in accordance with procedures approved by the Institutional Animal Care and Use Committee of the University of California Irvine.

### Animals and surgical procedures

Experiments were performed on white and wild‐type axolotls (*Ambystoma mexicanum*) measuring 12−15 cm snout to tail tip that were spawned at the University of California Irvine or at the Ambystoma Genetic Stock Center at the University of Kentucky. The animals were maintained in 40% Holtfreter's solution and were anesthetized prior to any procedure in a 0.1% solution of MS222 (ethyl 3‐aminobenzoate methanesulfonate salt, Sigma, St. Louis, MO), pH 7.4. After every surgical procedure, animals were placed on ice for 2 h to allow the wound to heal and to stabilize the placement of the nerve and ECM grafts, after which the animals were returned to 40% Holtfreter's solution.

### Induction of ectopic blastemas and limbs

The technique for inducing ectopic blastemas and limbs from wounds on the side of the limb has been described in detail previously (Endo et al. 2004; Satoh et al. 2007). Briefly, full‐thickness skin wounds on the anterior side of the limb were created by surgically removing a square of skin (2−3 mm on a side) from the anterior side of the stylopod (region of the humerus/femur), making sure that the underlying muscle was not damaged (Fig. 1A, D). An ectopic blastema was induced by surgically deviating the brachial nerve beneath the skin to bring the cut end of the nerve to the center of the skin wound (Fig. 1B, E). As demonstrated previously, to induce an ectopic limb, it was necessary to graft a piece of full‐thickness skin from the side of the limb that was opposite from the wound (Endo et al. 2004; Satoh et al. 2007). In this study we used two variations of this surgical procedure and grafted either decellularized skin (ECM graft, Fig. 1C, F) or artificial ECM in place of the full‐thickness skin. Wounds that were created on the posterior side of the arm responded to a deviated nerve and/or skin graft the same as anterior wounds (Endo et al. 2004). However, because of the anatomy of the limb, the deviated nerve often became retracted away from the wound site when the animal recovered from anesthesia and began swimming. We therefore focused on the response of anterior wounds to ECM‐mediated signaling. Data on the frequency of induction of blastemas and/or limb pattern were analyzed by Fisher's exact test (Supporting File 1).

### ECM preparation

Cell‐free (decellularized) ECM grafts were prepared by rinsing 2 mm × 2 mm pieces of full‐thickness skin in Ca^+2^/Mg^+2^ free Hanks solution, followed by incubation in 2 mol/L urea for 15 min. Samples were then rinsed in phosphate‐buffered saline (PBS) and stored in PBS at 4°C until grafted (Schaller & Muneoka 2001). Mouse anterior or posterior hindlimb stylopod skin or limb bud ECM was derived from FVB/NJ mice (embryonic day 11.5 [E11.5]to postnatal day 9 [PN9]). Pilot experiments with ECM derived from C57BL/6J or NOD SCID mice (PN3−4) gave similar results, suggesting there is not mouse strain specificity. Heparan sulfate moieties were removed enzymatically by incubating decellularized ECM preparations in 0.008 IU/mL heparin lyase III (Seikagaku) for 4 h at 37°C, after which they were rinsed in PBS and stored in PBS at 4°C until grafted. Heparin lyase III catalyzes the eliminative cleavage of alpha‐*N*‐acetyl I‐sulfo‐d‐glucosaminidic linkage of both membrane bound and secreted heparan sulfate and produces disaccharides, and does not act on chondroitin sulfate, dermatan sulfate, hyaluronic acid, or heparin. Growth factors and other signaling molecules that were bound to the ECM grafts were removed by incubating decellularized ECM preparations in 1 or 2 mol/L NaCl for 4 h at room temperature, after which they were rinsed in PBS and stored in PBS at 4°C until grafted (Schaller & Muneoka 2001). Growth factors were incorporated into ECM grafts by incubating decellularized ECM preparations in 500 μg/mL FGF2 or FGF8 (R&D Systems) for 2 h at room temperature, after which they were rinsed in PBS and grafted into host wounds.

### Artificial ECM preparation

Artificial ECM was made by combining 100 μL of 2.5 mg/mL (low HS), 5.0 mg/mL (med HS), or 10 mg/mL (high HS) heparan sulfate (porcine intestinal mucosa; Sigma) with 1.2 mL of 5.0 mg/mL type I collagen (calf skin; Sigma). This solution was incubated overnight at 4°C to form a precipitate that was then pelleted by centrifugation (10,000 rcf for 15 min) and grafted (Schaller & Muneoka 2001). Fast Green FCF and India ink were used to visualize the graft and determine that it was still present in the wound site post‐grafting.

### ECM grafting

The host site was prepared by creating a full‐thickness wound and surgically deviating the brachial nerve as described above. A wound epithelium formed and covered the wound within 6−12 h (Endo et al. 2004). The next day (18−24 h post‐wounding) an incision was made through the uninjured skin adjacent to one side of the skin wound, and fine‐tipped forceps were used to create a tunnel underneath the wound epithelium into which the graft was placed. ECM grafts were inserted through the tunnel with forceps and positioned adjacent to but not in contact with the deviated nerve (Fig. 1C, F). As with ECM grafts, artificial ECM was grafted 18−24 h after the initial surgery to create a wound and deviate a nerve. Because artificial ECM was viscous and could not be physically manipulated with forceps, the grafting procedure was modified by making an incision along three sides of the initial wound, the wound epithelium was then lifted up, and the artificial ECM graft was placed adjacent to but not in contact with the deviated nerve. The wound epithelium was then repositioned and allowed to heal back in place.

### Histological analysis

For the purposes of the current study, the experimental endpoint for pattern induction was whole‐mount skeletal preparations stained with Alcian Blue and Alizarin Red for samples collected 3 months after grafting at which point the final pattern was complete. We did not analyze the expression of marker genes (e.g., Hox, Meis, and shh) that are expressed at earlier time points that precede any visual verification of pattern induction. Skeletal elements in whole‐mount preparations were imaged and analyzed by fixing samples in 4% paraformaldehyde. They were then stained with 0.03% Alcian Blue 8GX (Sigma) and 0.01% Alizarin Red S (Sigma), and cleared with decreasing graduations of potassium hydroxide (KOH) and increasing graduations of glycerol.

### Reverse‐transcription polymerase chain reaction (RT‐PCR)

RNA was collected using an RNeasy Mini Kit (Qiagen). cDNA was reverse transcribed with Transcriptor (Roche) for subsequent PCR with goTaq Green PCR Mix (Promega). After 25 cycles (30 sec at 95°C, 30 sec at 58°C, 30 sec at 72°C) PCR products were separated by electrophoresis and visualized with ethidium bromide. To design primers, EST sequences of heparan sulfate sulfotransferases were retrieved from the Ambystoma Database (http://www.ambystoma.org/) according to homology to known full sequences using tblastx. Primer3 was used to create primer sequences that generate 150−200 base pair amplicons. EF1α was used as the normalizing expression control (Satoh et al. 2007).

The axolotl primers were as follows: HS2ST1, forward 5′‐TGCGCTTAGCCAAAGATTCCGCG, reverse 5′‐AGGAAAAGCATTGCCACCCCGA; HS3ST1, forward 5′‐TGCCGGCGTAACACAAACTAGCG, reverse 5′‐GGGACCGCTGTTTGCACGAGT; HS3ST2, forward 5′‐TGGTGGTGCGAAACCCGGTT, reverse 5′‐TTTCACCGGCTGGGTCCGTG; HS3ST3B1, forward 5′‐CCGCCAATGTGTCGTCATCGTCG, reverse 5′‐TTGGGCGGCAGAGAGGCATTC; HS3ST4, forward 5′‐TTGTGACCAAGGACGCCCCAAAG, reverse 5′‐TGTCTATCAAGCCGGTGCTGGCA; HS6ST1, forward 5′‐ACGCCTGACCCACACTACGTCA, reverse 5′‐CCAGGCGCACATTTTGTACCAGGT; NDST1, forward 5′‐CCTCAGCGTCTTTGTCTCCGCA, reverse 5′‐AGGGGTCCGTGCGGGATGAATC; NDST2, forward 5′‐CACACGCATGAAGGTGTCCGATGT, reverse 5′‐TCTGCTCTGCCAGGACAGTGACA; NDST3, forward 5′‐GAAGCTTGCCCTGTCTCTTGATCGG, reverse 5′‐AGAACTCATCCGACCGACCCCA; NDST4 forward 5′‐AAAACTGGCACCACGGCACTGT, reverse 5′‐GCGCCAACTCGTTTTGGGGCTT; EF1α, forward 5′‐AACATCGTGGTCATCGGCCAT, reverse 5′‐GGAGGTGCCAGTGATCATGTT.

The mouse primers were as follows: NDST1, forward 5′‐GCTTCCCAAAGCTTCTTATC, reverse 5′‐AAGAACTCCATGTACCAGTC; NDST2, forward 5′‐CTGCTGATTGGTTTCAGTCTTGT, reverse 5′‐CCACTGCTACTACAGTCTCCC; HS6ST1, forward 5′‐CGGACCCACATTACGAGAAAA, reverse 5′‐GATTGGGCCGATAGCAGGTG; HS2ST1, forward 5′‐TATGATGCCGCCCAAGTTG, reverse 5′‐CTGTTCAATTTCTCGGACTTCGT; HS3ST1, forward 5′‐CCCAGCTTGTGCATTCCCA, reverse 5′‐TGTGGAACCATTGGATGCTGT; HS3ST2, forward 5′‐GTGGACGTGTCTTGGAACG, reverse 5′‐CACTGACGAAGTGGATCTGGG; HS3ST3a, forward 5′‐ACCGGGATTTGATGCCCAG, reverse 5′‐GTCTGCGTGTAGTCCGAGAT; EF1α, forward 5′‐CAACATCGTCGTAATCGGACA, reverse 5′‐GTCTAAGACCCAGGCGTACTT; GAPDH, forward 5′‐TGTGTCCGTCGTGGATCTGA, reverse 5′‐TTGCTGTTGAAGTCGCAGGAG.

### RNA *in situ* hybridization

RNA *in situ* hybridization was performed on paraffin‐sectioned axolotl limb tissues as previously described (Lee & Gardiner 2012). Digoxigenin (DIG) labeled antisense RNA probes for axolotl HS3ST1, HS6ST1, and NDST2 were used to perform *in situ* hybridization. RT‐PCR was performed to amplify the sequences for antisense RNA probes with gene‐specific PCR primers. The specific PCR primers for the individual genes were as follows: HS3ST1, forward 5′‐GATGGAAGAGAGAGATGCTTGC, reverse 5′‐CGTGCGGTGTTATCAAGAGATG; HS6ST1, forward 5′‐GTCTCTACCACAAAGGTGGAAC, reverse 5′‐TCTAGAAGATGGCAAGGGTAGG; NDST2, forward 5′‐CACTCAAAGCAAACTGAGGACC, reverse 5′‐GCAGGATGCATAGTGAGGAAAG.

All template clones were subcloned into the pCRII vector (Invitrogen). The RNA probes were synthesized with the DIG RNA Labeling Kit (Roche) according to the manufacturer's protocol using T7 or Sp6 RNA polymerases. For tissue sample collection, tissues were collected and fixed in MEMFA (0.1 mol/L MOPS, pH 7.4, 2 mmol/L ethylene glycol tetraacetic acid, 1 mmol/L MgSO_4_, 3.7% formaldehyde), skeletal tissues were decalcified in Decalcifier I, dehydrated with an ascending series of ethanol (25%, 50%, 75%, and 100%), cleared in xylene, and embedded in paraplast. Paraffin sections were cut at 6 μm thickness. Sections were treated with 7.5 μg/mL of Proteinase K (Invitrogen) for 20 min at 37°C, refixed with 4% paraformaldehyde, and then hybridized with antisense RNA probes at 60°C overnight. After hybridization, sections were washed with buffer #1 (formamide:water:20× SSC = 2:1:1), then with buffer #2 (5:4:1), and blocked with 2% blocking reagent (Roche) in TBST for 30 min. The sections were then incubated with 1:2000 diluted alkaline phosphatase conjugated anti‐DIG antibody (Roche) overnight at 4°C. The color staining reaction was performed using BM purple (Roche) as a substrate for alkaline phosphatase.

### Glycosaminoglycan sulfation analysis

The level of glycosaminoglycan (GAG) sulfation was quantified by the dimethyl‐methylene blue assay (Blyscan Assay Kit, Biocolor, UK). Anterior and posterior salamander limb skin samples were collected from the stylopod region of uninjured limbs with 2 mm biopsy punches. Statistical significance was tested by Student's *t*‐test.

## Supporting information

Additional Supporting Information may be found in the online version of this article at the publisher's website:
reg240‐sup‐0001‐Supporting‐File‐1.xlsx
